# The Effect of Hearsay on Speaker Commitment: An fEMG Study

**DOI:** 10.1162/OPMI.a.382

**Published:** 2026-08-21

**Authors:** Harriet Yates, Bob van Tiel, Peter de Swart, Corien Bary

**Affiliations:** Faculty of Philosophy, Theology, and Religious Studies, Radboud University Nijmegen, Nijmegen, The Netherlands; Faculty of Arts, Radboud University Nijmegen, Nijmegen, The Netherlands

**Keywords:** commitment, evidentiality, hearsay, pragmatics, facial electromyography (fEMG)

## Abstract

When reporting speech, speakers not only relay information, but may also signal their degree of commitment to that information. This study aims to gather reliable empirical data on the role of so-called reportative evidentials (like ‘I heard that Jack is a bachelor’) in impacting the degree of perceived speaker commitment (here, their commitment to the truth of Jack being a bachelor). To measure perceived speaker commitment we use the physiological method of *facial electromyography* or fEMG. We show how this method can be used to trace perceived norm violations in conversation, from which we can then derive the commitments attributed to the discourse participants. In addition to comparing assertions with and without the use of reportative evidentials, we zoom in on the differences between two common reportative constructions: embedding (‘I heard that Jack is a bachelor’) versus parenthetical (‘Jack is a bachelor, I heard’). In the literature there is controversy as to whether these two constructions have the same effect on speaker commitment. Results show that the use of reportative evidentials indeed lowers (but does not fully remove) perceived speaker commitment, but do not provide evidence that the two constructions have different effects. The findings can be understood within discourse frameworks that both model the contents of what is said and the degree of commitment to these contents.

## INTRODUCTION

When we speak, we not only present information for discussion, but we may also signal our degree of commitment to that information. Evidentials, linguistic expressions that we use to signal the kind of evidence that we have for our claims (visual or hearsay for example), are often argued to modulate the expressed degree of commitment (e.g., Faller, 2002). This study aims to gather reliable empirical data on the role of so-called reportative evidentials (like ‘I heard that Jack is a bachelor’) in impacting the degree of perceived speaker commitment (here, their commitment to the truth of Jack being a bachelor).

When making an assertion, a speaker may choose a plain form:

(1)  Jack is a bachelor.

Alternatively, a speaker may choose to linguistically mark the kind of evidence from which their information was obtained. Thus an assertion of (2) indicates not only the propositional content that Jack is a bachelor, but additionally communicates that the speaker has hearsay evidence towards the claim.

(2)  I heard that Jack is a bachelor.        [EMBEDDING]

A prominent question in the literature is whether adding the reportative evidential ‘I heard’ reduces the perceived commitment of the speaker to Jack being a bachelor in (2) compared to (1). Below we list three possible answers that can be distinguished. Here and elsewhere in this paper, the phrase ‘commitment to the (truth of the) proposition/content expressed’ should be taken as concerning the reported content (in (2) *that Jack is a bachelor*) rather than the reportative component (in (2) *that the speaker has heard that Jack is a bachelor*).

(a)  The hearsay expression altogether removes the speaker’s commitment, since the speech act merely commits the speaker to having heard someone say the propositional content, and not to the truth of that content. This is the position that Faller (2002) advocates for the reportative evidential ‘-si’ in Cuzco Quechua, for example, and Murray (2014) for ‘I heard that …’ in English.
(b)  The speaker remains consistently committed to the proposition, and the reportative evidential merely provides further information about the source of evidence. This is what Hunter (2016) for example claims for English parenthetical ‘…, she said’.
(c)  The reportative evidential reduces, but does not altogether remove, the speaker’s commitment to the truth of the proposition. Koev (2021) advocates this position for English embedding ‘She said that …’ and parenthetical ‘… , she said.’ This answer suggests that commitment is a matter of degree, rather than an all-or-nothing notion.

In the present study, we seek to develop a method to be used to arbitrate between these three possibilities, which we apply to Dutch ‘ik hoorde’ (‘I heard’) as a first case.^1^ The same method can be applied to other constructions and languages in later studies.

In addition to comparing assertions with and without ‘I heard’, we also investigate whether the answer depends on the syntactic prominence of the expressed proposition. To illustrate, compare (2) with (3):

(3)  Jack is a bachelor, I heard.        [PARENTHETICAL]

In (2), the clause expressing the propositional content that Jack is a bachelor is *embedded* with respect to the reportative main clause. By contrast, in (3) the propositional content forms the main clause, and due to its prominence, may perhaps be interpreted as the ‘main point’ of the utterance (Simons, 2007). The reportative evidential is here included as ‘just’ a *parenthetical* clause. As a consequence, several authors have expressed the intuition that parenthetical constructions lead to a greater speaker commitment to the expressed content than embedding ones.^2^

Thus, the purpose of this study is twofold. First, we investigate how the presence of the reportative evidential ‘I heard’ impacts the perceived speaker commitment to the truth of the expressed proposition. Second, we investigate whether this effect is modulated by the syntactic prominence of the clause expressing this proposition.

To answer these questions of commitment, we study the felicity of denying the expressed proposition. It is generally infelicitous as a speaker to deny propositions to which one has previously committed: ‘*p*, (but) not *p*’ or ‘I know that *p*, (but) not *p*’. One can take felicity of such denials as a starting point for a test of commitment, used in, for example, Bonalumi et al. (2023) and Weissman 2022), but we find the idea already in Asher (2000), Jackendoff (1972), and many others. In the case of the simple assertion in (1), it would be decidedly odd for the speaker to continue with ‘But he’s married.’ However, what if the speaker continues in the same way after an utterance of (2) or (3)? Here intuitions already become much less clear. This is a more general complication: judgments regarding speaker commitment are often very subtle and nuanced (Mazzarella et al., 2018).

For this reason, we choose to take an experimental approach to the felicity of denying the expressed proposition. Specifically, we use facial electromyography (fEMG), a physiological method measuring facial muscle activity. This technique enables researchers to precisely target specific muscles, which can be mapped onto participants’ emotional states, thereby providing information about the valence and intensity of their affective responses (Ekman et al., 1972; van Boxtel, 2010). There is a large literature showing that the *corrugator supercilii* muscle (the inner-eyebrow muscle used for frowning)—whose activation is associated with negatively valenced affective responses—becomes active in cases of norm violations (Bartholow et al., 2001; ’t Hart et al., 2018; van Berkum et al., 2023). In previous work, we have shown that this extends to the communicative norm that one should not contradict their own prior commitments (Yates et al., 2025). In that study, we showed that the corrugator becomes highly active when participants read about a speaker who denies an earlier asserted proposition, in contrast to when the same proposition was posed as a question. That is, an observer frowns when witnessing a commitment violation. This study suggests that fEMG offers a reliable way to measure the assignment of speaker commitment in communication.

The fEMG methodology offers a number of important benefits for research in the field of discourse commitments:

1. fEMG allows precise, muscle-specific measurement, enabling researchers to trace the emotional response of indignation triggered by a commitment violation (Bartholow et al., 2001).
2. fEMG offers high sensitivity, detecting subtle facial muscle activity (including nonvisible contractions), capturing responses before or without overt expressions (Rutkowska et al., 2024; van Boxtel, 2010).
3. Related to the sensitivity of the measure, in the linguistic literature certain expressions or contextual factors are often said to *reduce the degree* of commitment. fEMG offers the capacity to capture this ‘gradable’ nature of commitment, tracking responses across a continuous scale of muscle activation.
4. fEMG provides an implicit measure of immediate, spontaneous affective response (even preconscious and involuntary, Cacioppo et al., 2000), capturing reactions that could otherwise be lost when participants must reflect or articulate explicit judgments. This reduces the potential impact of self-modulation or social desirability bias, wherein participants may consciously or unconsciously adjust their true responses in order to align with perceived expectations or to present themselves in a favourable light (Crowne & Marlowe, 1960; Dodou & de Winter, 2014).
5. fEMG delivers high temporal resolution, with detectable activity known to emerge as early as 100–200 ms from stimulus onset, allowing affective responses to be tracked as they unfold, and enabling the use of fine-grained analyses such as time-course modeling to reveal nuanced dynamics (e.g., ’t Hart et al., 2018; van Boxtel, 2010).
6. fEMG, through baseline-normalized microvolt activity, offers an objective and standardized metric that accounts for individual differences (e.g., in physiological capacity, some people simply have stronger corrugator muscles than others). This normalization enables reliable inter- and intra-participant comparisons, ensuring that a participant with weaker absolute responses is not less valuable to the overall picture (van Boxtel, 2010; van Boxtel & van der Graaff, 2024).

In sum, given subtleties of intuitions, measuring the perceived commitment that conversational partners attribute to a speaker’s utterance benefits from the use of a highly sensitive, implicit measure such as fEMG.

One remark about the theoretical framework before we proceed. There are two main stances on what the primary effect of assertions is. In this paper we opt for a commitment-based framework in which a plain assertion of (1) has as its primary consequence that the speaker becomes committed to the truth of the propositional content (e.g., Hamblin, 1971). There is also an alternative take on assertions in which they primarily convey beliefs and intentions. The most explicit defenders of this second approach are Bach and Harnish (1979), who claim that by asserting that *p* the speaker expresses the belief that *p* and the intention that the hearer believe that *p*.

In the philosophy of language, where this is a central debate, the social and normative character of commitments gets much attention. We find this approach for example in Brandom (1983); Geurts (2019); Hamblin (1971); Krifka (2015); MacFarlane (2011); Searle (1976). Following this tradition we define a commitment in general as an obligation to others to act in certain ways. By our linguistic acts, we create expectations for our conversational partners, who in turn are entitled to expect us to act consistently with our prior commitments. Commitments thus constrain how we *should* behave. In the specific case of assertions, our commitments concern the truth of the contents that we express. While opinions differ on what exactly this entails (e.g., Brandom (1983) argues this is justificatory responsibility to the truth when challenged, whereas MacFarlane (2011) argues that this is too strong), at the very minimum it entails that one should not continue by asserting something that is inconsistent with an earlier assertion, and that is what we will make use of in this study.

In more empirical linguistic work the notion of commitment is used in the analysis of many linguistic phenomena (discourse particles, evidentiality markers, intonation) and the idea of a conversational scoreboard of commitments is, for example, central in Faller (2019), Farkas and Bruce (2010), Gunlogson (2008), Krifka (2015, 2023), Portner (2018), and much subsequent work. The difference between (private) beliefs and (public) commitments is often mentioned, but the normative dimension does not always get much attention. Still, in a light version this dimension is present here, too: a speaker should not contradict themselves.

Nothing in our present study hinges on one’s position within this debate. While it is in general important to distinguish beliefs and commitments (e.g., Bary, 2025; Geurts, 2019), they often go hand in hand, and what differs is just who is in the driver’s seat and who follows in the back seat (Geurts, 2019). Readers who favor a belief-and-intention view on assertion may thus want to reinterpret our question of commitment in terms of certainty (‘Is a speaker who includes a marker of hearsay evidence perceived as expressing greater uncertainty compared to the plain assertion?’) and the increased corrugator activity may then be a sign of the emotion of confusion rather than indignation (Durso et al., 2012). So, our study cannot be used to differentiate between the commitment-based and belief-and-intention-based accounts of communication.

Thus having established our motivations and the theoretical embedding, the remainder of this paper is structured as follows: in the next section we describe our study in more detail. Afterwards, we present the results, followed by a discussion of their implications. We conclude with some thoughts about future applications of this methodology in relation to discourse commitments.

## EXPERIMENTAL DESIGN

### General Set-Up

In this section we will present the design of the experiment conducted to investigate the effect of reportative evidentials on perceived speaker commitment through the methodology of fEMG. The experiment was set up as follows: participants read short descriptions of social interactions between two people, in Dutch. While reading, their corrugator activity was continuously recorded, with activity during the critical segment to be compared with baseline muscle activation that was measured at the start of each trial while participants saw a distractor image. From this we may derive a participant’s implicit judgment of the given items.

The primary manipulation of the items were three types of declarative sentences, as shown in Table 1. The first are sentences like ‘Jack is a bachelor’, without any markers that may hedge the speaker’s commitment such as evidentials or discourse particles. We will refer to this type as ‘plain assertion’, indicating the speech act performed. We subsequently adapted these sentences into two syntactic constructions with reportative evidentials, resulting in sentences with ‘I heard’ in embedding and parenthetical form.

**Table 1.** (Translated) example items for all conditions

**Story introduction**
Valerie and Louis are chatting about their friend Jack.
**Experimental Conditions**
plain assertion–incongruent
Jack is a bachelor. But he is married.
embedding ‘i heard’–incongruent
I heard that Jack is a bachelor. But he is married.
parenthetical ‘i heard’–incongruent
Jack is a bachelor, I heard. But he is married.
**Control Condition**
plain assertion–congruent
Jack is a bachelor. He is single.

Uttering these initial sentences may lead to a conversational commitment of the speaker to the truth of the expressed proposition, here that Jack is a bachelor (and this may differ per condition—this is exactly what we aim to investigate through this experiment). On the next screen seen by participants, they read that the same speaker then contradicts this proposition with an incongruent follow-up assertion. Figure 1 shows the central part of the on-screen presentation that a participant sees.

**Figure 1.**
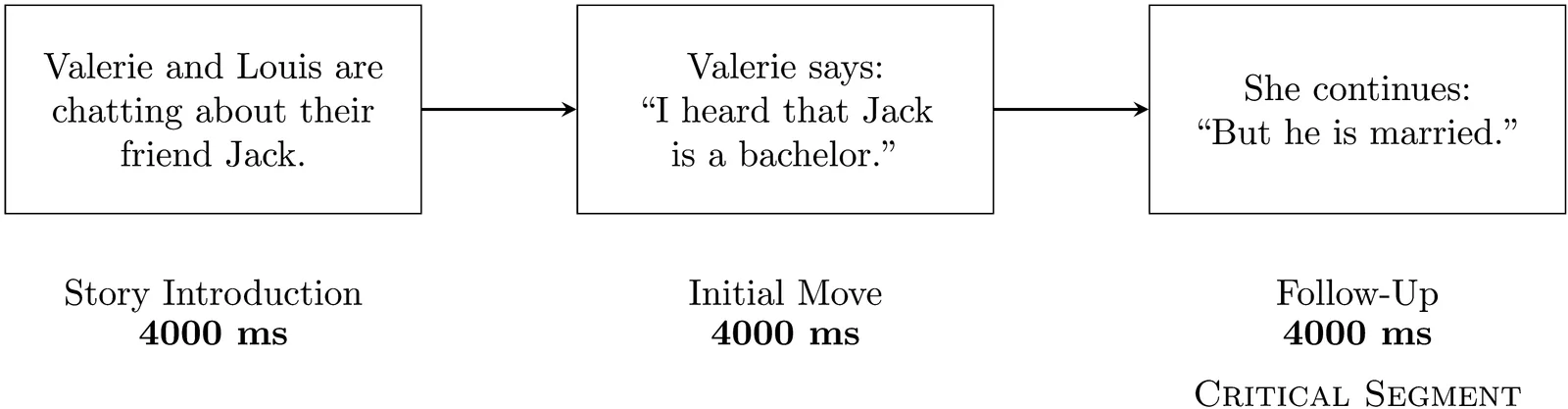
A flowchart showing the central part of the on-screen presentation of a single item trial, here for an embedding ‘I heard’ initial move.

While corrugator activity is continuously recorded, it is this follow-up section that forms the critical segment: the corrugator activity here tells us how participants perceive the speaker’s continuation, with a greater corrugator activity indicating a greater perceived norm violation. From this we can derive how participants understood the initial move, i.e., to what extent—according to the participants in the experiment—the speaker took on a commitment to the expressed proposition (here, Jack being a bachelor). A greater perceived norm violation in the critical segment means participants understood the speaker as more strongly committed to the initial proposition.

For communicative ease, the labels for the conditions have two parts. The first refers to the initial move and the second to the follow-up: plain assertion–incongruent, for example, indicates that the initial move is a plain assertion which is followed up by an incongruent continuation. In addition to the three experimental conditions, we also included a plain assertion condition with a congruent follow-up. This control condition functions to show a speaker acting in line with their previous utterance, in which no discourse commitments are violated.

This brings us to our hypotheses. The speech act of a plain assertion is seen as a relatively uncontroversial way to take on commitments in communication in the literature; someone who makes an assertion takes on a commitment to the truth of the proposition they express (Brandom, 1983; Geurts, 2019; Hamblin, 1971; Searle, 1976). This commitment entails that the speaker will be taken to be violating a norm if they continue by asserting something that is inconsistent with the earlier asserted proposition. Reportative evidentials, by contrast, are often claimed to reduce the speaker’s commitment. On the basis of this, we hypothesize that (i) plain assertion–incongruent should elicit higher corrugator activation than the ‘I heard’ conditions, since in this case the speaker has undertaken the clearest and strongest commitment to their initial asserted propositions, making the subsequent contradiction a greater violation. The reportative evidential constructions should elicit lower corrugator activation.

Hypothesis (ii) concerns the possible modulation of commitment depending on the prominence of the expressed proposition in the initial move. Because commitment is expected to be higher when the proposition is presented in a main clause than when it is embedded under a reportative construction, we predict greater corrugator activity in the parenthetical ‘i heard’–incongruent condition, relative to the embedding one.

The purpose of including the plain assertion–congruent control is to show that the experimental conditions elicit a meaningful level of corrugator activation in comparison to a no-violation context. Explicitly, our Hypothesis (iii) is that the control condition will show minimal corrugator activation, significantly lower than all experimental conditions. The control condition also serves the additional purpose of a sanity check for the experimental set-up and practical execution; if substantial corrugator activity were observed, we should question the validity of the data gathered throughout the experiment.

### Participants

The study recruited 66 participants (52 female, 12 male, 2 other; age range: 18–71 years; mean age: 26) via the centralized university recruitment database of Radboud University. Participants were compensated with retail vouchers or academic credit. All participants were adult native speakers of Dutch, with no known reading or learning disorders, and with normal or corrected-to-normal vision. Participants with a history of Botox injections were not permitted to participate, as this can inhibit the motor action of muscles targeted in fEMG. The study was approved by Radboud Ethics Assessment Committee for Humanities. Informed consent was obtained from all participants prior to participation.

### Sample Size Justification

The sample size was determined primarily by practical constraints, including the financial resources available for participant compensation, the time required for recruitment and testing, and the availability of testing facilities. Individual testing sessions lasted approximately 90 mins, and recruitment took approximately three months to complete. Nevertheless, the resulting sample is large compared to similar previous studies (e.g., Bartholow et al., 2001; Durso et al., 2012; Thompson et al., 2016).

To provide an indication of the sensitivity of the present setup, we conducted a simulation-based sensitivity analysis using data from the proof-of-method study discussed above. Focusing on the comparison between congruent and incongruent assertions, the analysis indicated that, under the variance structure observed in that study, effects of approximately 6 percentage points in baseline-corrected corrugator activity could be detected with 80% probability in a sample of 41 participants. The present study analysed data from 57 participants (after exclusions, see below). Although the design and materials differed from those of the proof-of-method study, this benchmark suggests that the present study was likely sensitive to corrugator effects of a similar magnitude.

### Materials

We designed 64 item sets, each appearing in the four conditions illustrated in Table 1, following a Latin square design. Each trial consisted of a distractor image (3,000 ms), a brief story introduction (4,000 ms), a speaker’s initial discourse move (4,000 ms), and their follow-up move (4,000 ms). Figure 2 gives again an example of one trial as presented to a participant, this time in its full form. The distractor segment shown per-trial serves to provide a neutral pause in which the corrugator may relax, from which we calculate the baseline muscle activity. The condition manipulation occurs in the initial move slide, while the presentation of the (in)congruent follow-up is the critical segment.

**Figure 2.**
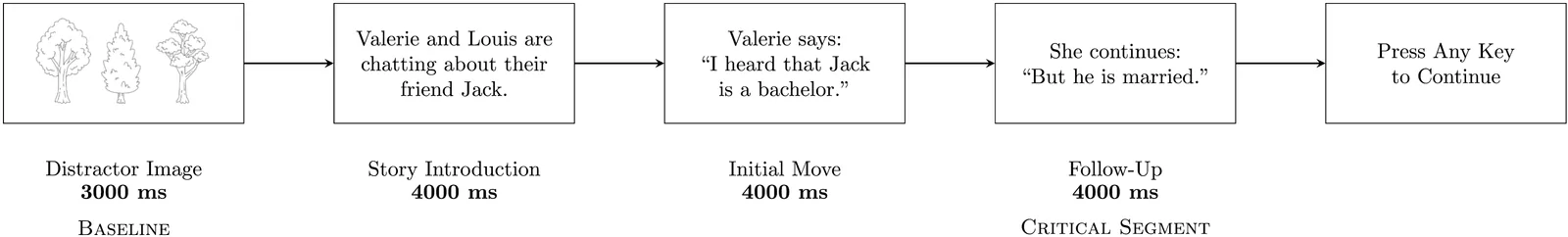
A flowchart showing the on-screen presentation of a single embedding evidential item trial (experimental item with incongruent follow-up). The distractor image here is for illustrative purposes only. The segments relevant for analysis are marked as Baseline and Critical Segment.

Finally, 32 filler item sets were included for two purposes. One was to reduce predictability and distract participants from the experimental manipulations. The second was to ensure that any observed corrugator activity could indeed be attributed to a commitment violation, rather than to the syntactic structure (embedding or parenthetical) or to the presence of the contrastive marker ‘maar’ (‘but’ in English) itself. The contrastive marker was included in the experimental items (see Table 1) to mirror examples in the literature on reportative evidentiality and commitment: even authors who claim that reportative evidentials remove all commitment still consider a contrastive marker necessary in these cases for a coherent discourse (see discussion in, e.g., AnderBois, 2014). For that reason, the fillers consisted of 16 embedding and 16 parenthetical ‘I heard’ constructions (Table 2), balanced such that, within each syntactic construction, half contained the contrastive marker and half did not. We expected minimal activity in the fillers since no commitments were violated.

**Table 2.** (Translated) example items for filler conditions. This example is representative only, as the content of the filler conditions do not appear in all conditions

**Story introduction**
Natasha and Jonathan are chatting about hobbies.
**Filler Conditions**
embedding ‘i heard’–congruent (no contrastive)
I heard that Stijn plays a team sport. He plays volleyball.
embedding ‘i heard’–congruent (with contrastive)
I heard that Stijn plays a team sport. But he also swims.
parenthetical ‘i heard’–congruent (no contrastive)
Stijn plays a team sport, I heard. He plays volleyball.
parenthetical ‘i heard’–congruent (with contrastive)
Stijn plays a team sport, I heard. But he also swims.

All items began with a story introduction, a brief contextualizing sentence which establishes the social nature of these conversations, for which two people were present. This was important to the research question, as to undertake a commitment is a public, shared act. Were this social context not established, participants may deem the follow-up incongruence to be permissible and of less consequence. Despite the involvement of two social actors, the items emphasized that it was the same speaker uttering both the initial speech act and the follow-up (there would be no commitment violation if it were another speaker who contradicted the initial assertion). Traditionally-gendered Dutch names were used so that referential pronouns could be used clearly within the items when necessary.

As fEMG recording readily detects emotional reactions to items, the content of all items was designed under limitations. In particular, strongly valenced content is known to trigger corrugator activation, independently of the evaluation of the initial move which we hoped to elicit in this experiment (’t Hart et al., 2018). To avoid such effects, all items used non-emotive language as far as possible, and did not describe situations which we would take to be social norm violations, outside of the communicative norm violation that we are interested in (i.e., that of acting against one’s earlier commitments).

### Procedure

The experiment took place in the Center for Language Studies laboratory at Radboud University. The fEMG signal was recorded using Biopac hardware (MP150 module and EMG100C Electromyogram Amplifier) and Biopac’s Acqknowledge software (BIOPAC Systems, 2024). Three surface electrodes were attached to the participant; one acting as the ground (a location on the hairline with minimal palpable muscle activity), and two electrodes at bipolar corrugator recording sites (above the eyebrow in line with the pupil and on the inner side of the eyebrow). The electrode placement was based on conventions taken from the Facial Action Coding System (Ekman et al., 1972) and the guidelines for EMG developed by Fridlund and Cacioppo (1986). The electrodes were automatically referenced within the Acqknowledge software system. The electrode sites were gently cleaned with abrasive gel and alcohol to lower inter-electrode impedance. This electrical impedance (a measure of the opposition to electrical flow, in kΩ) was measured per participant prior to recording; when impedance is too high this can result in a noisy signal and the loss of meaningful fEMG signal. The fEMG signal was continuously recorded, in muscle unit action potentials (*μ*V).

The trials were presented to the participants using OpenSesame software (Mathôt et al., 2012). Two preparatory trials familiarized the participant with the procedure. Then each participant read 96 pseudo-randomized vignettes (64 experimental and control items (16 per condition) and 32 fillers), presented line-by-line in time-locked segments. As previously mentioned, each trial was preceded by a 3,000 ms distractor image (showing a sunlit path through a forest). This segment was used to calculate baseline corrugator activity to benchmark corrugator activity during the stories. An image was used instead of, e.g., a fixation cross because the former is thought to elicit a more relaxed level of baseline corrugator activity (Fridlund & Cacioppo, 1986; ’t Hart et al., 2018).

Although the on-screen presentation of each segment within a given trial was time-locked, advancement to the next trial was self-paced (in the format “Press any key to continue”). Participants were encouraged to take breaks whenever necessary, in an effort to avoid fatigue. Participants were asked to read the items silently (as opposed to out loud) to control for possible muscle interference from speaking. To ensure that the participants were sufficiently attending to the items, twelve true/false comprehension questions about the story introduction were randomly interspersed amongst the items. Participants were to be excluded if they made more than two errors on these comprehension questions.

### Participant Exclusion

We used the same exclusion criteria as the proof-of-method study. Thus, nine participants were excluded from the final analysis. Eight of those participants had unresolvable high electrical impedance (greater than 40 kΩ) on the electrodes at the time of recording. One participant was excluded because their mean activation in the critical follow-up segments deviated more than 2.5 standard deviations from the overall group mean. No participants were excluded due to the comprehension questions, as all correctly answered at least 10 out of 12 questions. This resulted in 57 data sets for analysis (43 female, 12 male, 2 other; age range: 18–71 years; mean age: 26).

### Data Preparation

Data files were imported into Matlab for preprocessing (The MathWorks Inc., 2026). We followed recommended preprocessing steps as laid out by van Boxtel (2010) and which were used by our lab in the earlier proof-of-method experiment (Yates et al., 2025).

The raw fEMG signal was first filtered with a 20–500 Hz band-pass filter and a 50 Hz notch filter to eliminate possible mains power electrical interference from the recorded signal. The signal was rectified and the key segments (i.e., the 3,000 ms distractor segment and 4,000 ms critical follow-up segment) were isolated. The 3,000 ms distractor segment was smoothed using the LOESS method and the mean absolute value was calculated per-trial, in order to obtain an artifact-free baseline measure of corrugator activity. The 4,000 ms follow-up segments were divided into 40 100ms bins, an interval which provides optimal temporal resolution to track rapid dynamic facial muscle activity while largely avoiding random error (van Boxtel, 2010; van Boxtel & van der Graaff, 2024). The amplitude in the follow-up segment was expressed as a percentage of the baseline mean absolute value for that trial. This method accounts for both intra- and inter-participant variation in fEMG amplitude (van Boxtel & van der Graaff, 2024). These data were then exported from MATLAB to R for statistical analysis (R Core Team, 2025).

## RESULTS

### Main Analyses

Although the experiment was not preregistered, all analysis plans were made prior to data collection, unless stated otherwise.

To reiterate the hypotheses to be tested, as outlined previously:

(i)  Corrugator activation in the critical segment will be higher for the plain assertion–incongruent condition than for the ‘I heard’ items.
(ii)  Corrugator activation in the critical segment will be higher for parenthetical constructions compared to embedding ones.
(iii)  Corrugator activation in the critical segment will be lower for the plain assertion–congruent control condition than for all experimental conditions.

Figure 3 shows a timeplot of corrugator activation throughout the full trial period, expressed as a percentage of per-trial baseline activation in the distractor segment (when participants saw the image of a sunlit forest). Figure 4 shows mean corrugator activation per condition over the entire critical follow-up segment, while Figure 5 again shows a timeplot for the same segment.

**Figure 3.**
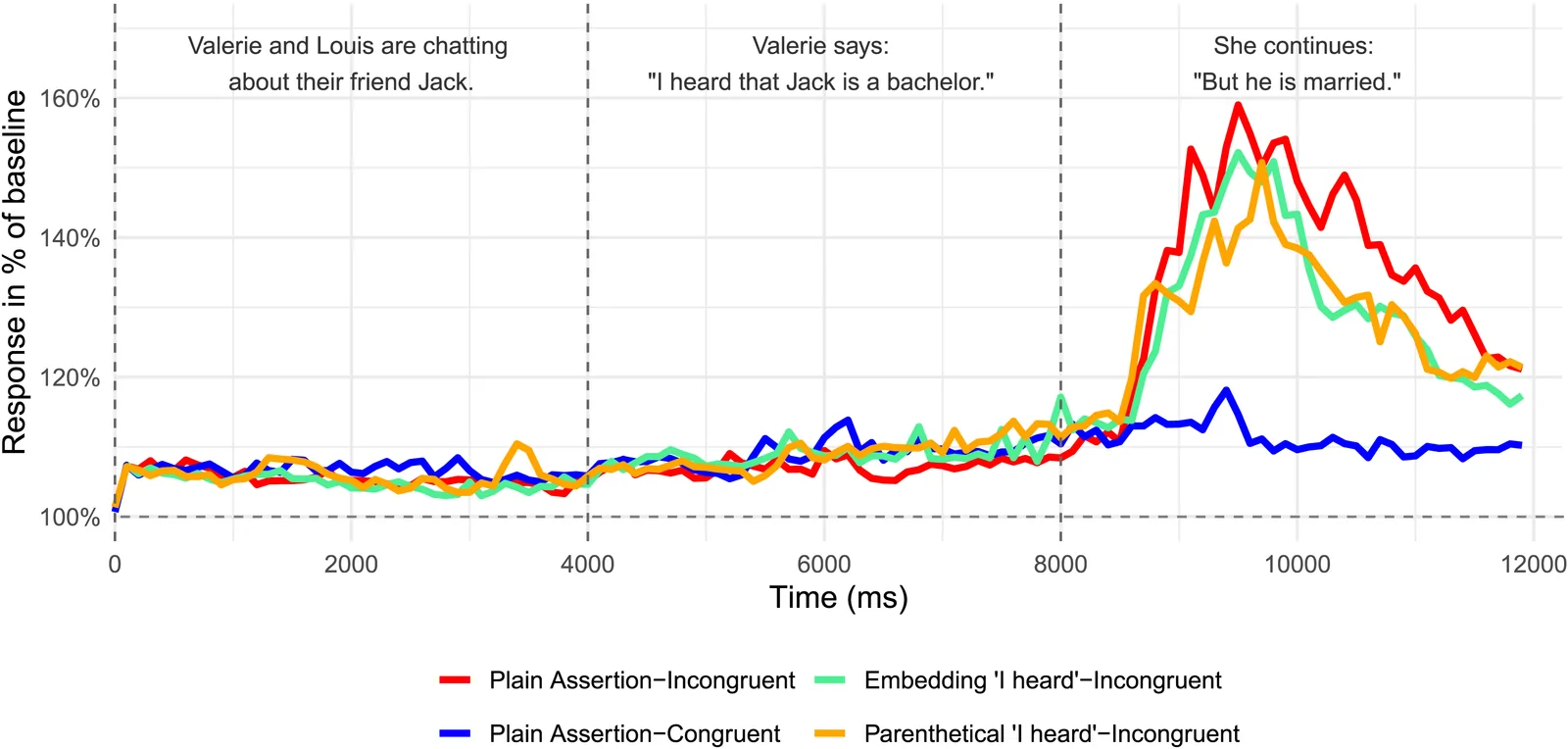
Time course of participants’ corrugator response, expressed as a percentage of per-trial baseline activation, across the full trial. An example item is shown to correspond with the data.

**Figure 4.**
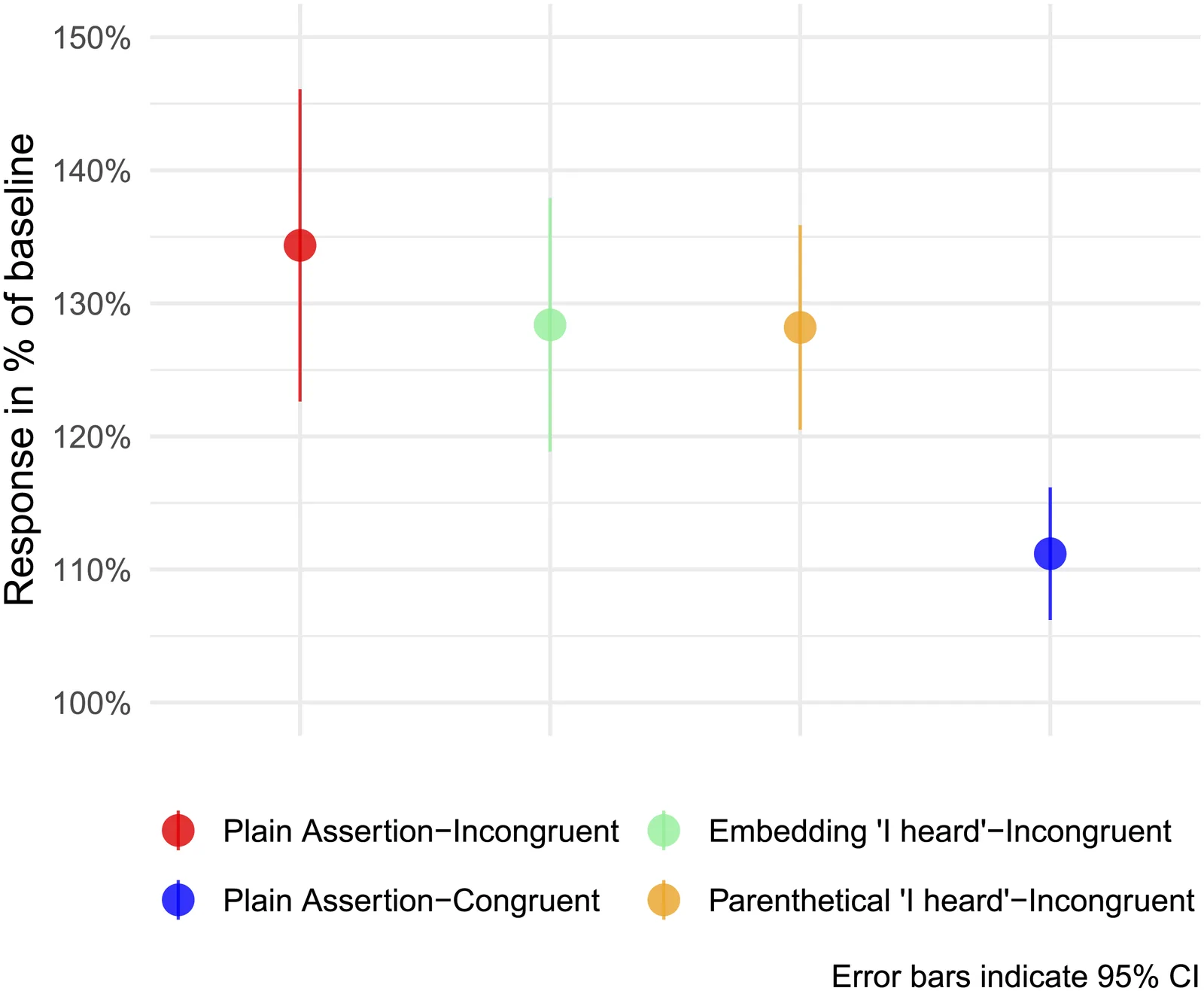
Mean corrugator activation in the critical segment.

**Figure 5.**
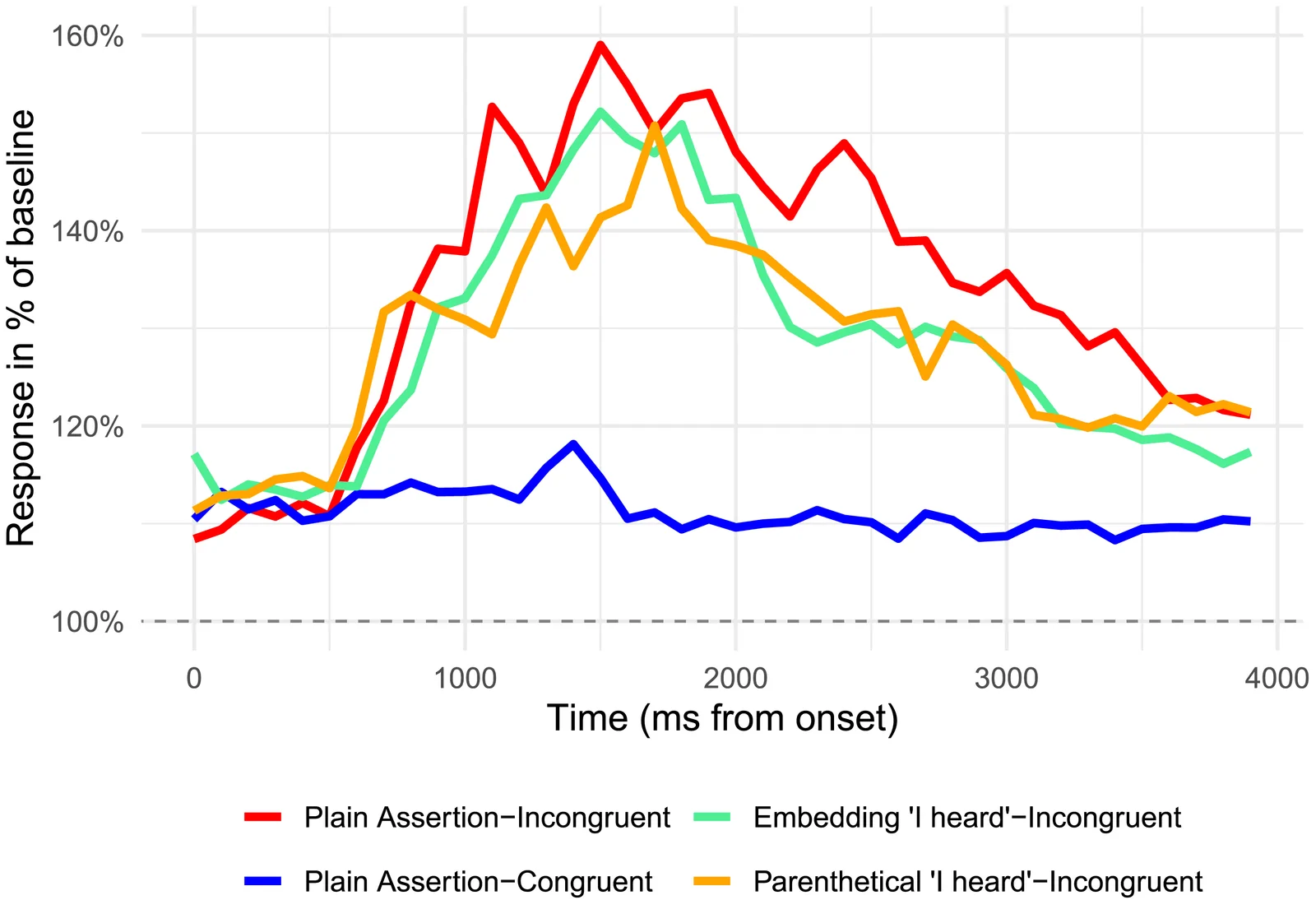
Timeplot of corrugator activation in the critical segment.

In the plain assertion–incongruent condition, mean activation during the critical segment was 134.4% of the baseline, it was 128.4% in the embedding ‘i heard’–incongruent condition, 128.2% in the parenthetical ‘i heard’–incongruent condition, and 111.2% in the plain assertion–congruent control condition. So, descriptively, mean activation was highest in the plain assertion–incongruent condition and lowest in the plain assertion–congruent control condition.

We first tested Hypothesis (iii), which predicts lower corrugator activation in the plain assertion–congruent control condition than in the experimental conditions. Corrugator activation in the critical segment was indeed higher in the experimental conditions than in the plain assertion–congruent control condition. To determine if these differences were statistically significant, we fit a linear mixed-effects regression model predicting per-trial mean corrugator activation in the critical segment (expressed as a percentage of baseline), including fixed effects of condition and trial number, as well as random intercepts for participants and items. Statistical analyses were carried out using the ‘lme4’ package (Bates et al., 2015). Corresponding *p*-values were estimated using the Satterthwaite procedure as implemented in the ‘lmerTest’ package (Kuznetsova et al., 2017). The plain assertion–congruent condition served as reference level.

The results showed significantly higher corrugator activation in all three experimental conditions compared to the control condition. Thus, compared to the percentage of corrugator activation for the plain assertion–congruent control condition (111.2%), the plain assertion–incongruent condition elicited an estimated increase in mean activation of 23.17 percentage points (*SE* = 3.22, *t* = 7.19, *p* < .001), the embedding ‘i heard’–incongruent an increase of 17.19 percentage points (*SE* = 3.22, *t* = 5.34, *p* < .001), and the parenthetical ‘i heard’–incongruent an increase of 16.99 percentage points (*SE* = 3.22, *t* = 5.28, *p* < .001).

Hypotheses (i) and (ii) specifically concern the plain assertion–incongruent and two ‘I heard’ conditions. To test these hypotheses, we fit a linear mixed-effects regression model of the same form, again predicting per-trial mean corrugator activation in the critical segment (expressed as a percentage of baseline), with fixed effects of condition and trial number and random intercepts for participants and items. We applied custom Helmert-style contrasts to isolate two theoretically motivated comparisons: (a) plain assertion vs. the average of the embedding and parenthetical items, and (b) embedding vs. parenthetical.

The first contrast, targeting the effect of evidentiality markers together, was statistically significant (*b* = 6.10, *SE* = 2.91, *t* = 2.10, *p* = .036, 95% CI = [0.39, 11.81]). This indicates that the plain assertion–incongruent condition elicited reliably greater activation than the ‘I heard’ constructions. Thus, adding ‘I heard’, regardless of syntactic form (embedding vs. parenthetical), elicited less corrugator activity than the plain assertion–incongruent condition, confirming Hypothesis (i).

The second contrast compared the ‘I heard’ constructions and yielded no significant difference (*b* = 0.22, *SE* = 3.36, *t* = 0.06, *p* = .95, 95% CI = [−6.37, 6.80]). We therefore found no evidence that parenthetical constructions elicited greater corrugator activation than embedding constructions, contrary to Hypothesis (ii).

Trial number did not significantly predict mean activation for the experimental conditions (*b* = 0.02, *SE* = 0.05, *t* = 0.47, *p* = .64), which indicates that corrugator activation remained stable over the course of the experiment.

### Exploratory Analyses

#### Temporal Dynamics.

In addition to the main analyses, we carried out a number of exploratory analyses.

To better understand the difference between plain incongruent assertions and the two evidential conditions, we investigated their temporal dynamics using cluster-based permutation analyses. This method is designed for situations in which condition differences are assessed across many adjacent time points. Rather than evaluating each time bin separately, it identifies clusters of consecutive bins which exhibit a consistent effect, and assesses their significance using a permutation-based null distribution. This approach controls the family-wise error rate while remaining sensitive to temporally extended effects (Maris & Oostenveld, 2007).

Specifically, we conducted cluster-based permutation analyses on the 100 ms time bins in the critical segment using the ‘clusterperm.lmer’ function in R. For each of the three theoretically relevant pairwise comparisons, we fitted a linear mixed-effects model with baseline-corrected corrugator activation as the dependent variable, condition as a fixed effect, and random intercepts for participants and items. Cluster-level significance was assessed using the cluster mass statistic and 1,000 permutations. To control for the three planned comparisons, we applied a Bonferroni-corrected significance threshold of *α* = .017.

Comparing the plain assertion–incongruent and embedding ‘i heard’– incongruent conditions, significant clusters were observed at 2,200–2,800 ms and 3,100–3,600 ms (Figure 6). Comparing the plain assertion–incongruent and parenthetical ‘i heard’– incongruent conditions, significant clusters were observed at 1,200–1,700 ms, 2,400–2,600 ms, and 3,100–3,500 ms (Figure 7). In both comparisons, the largest clusters occurred towards the end of the critical segment. This pattern suggests that the evidential conditions returned toward baseline more rapidly than the plain incongruent assertions.

**Figure 6.**
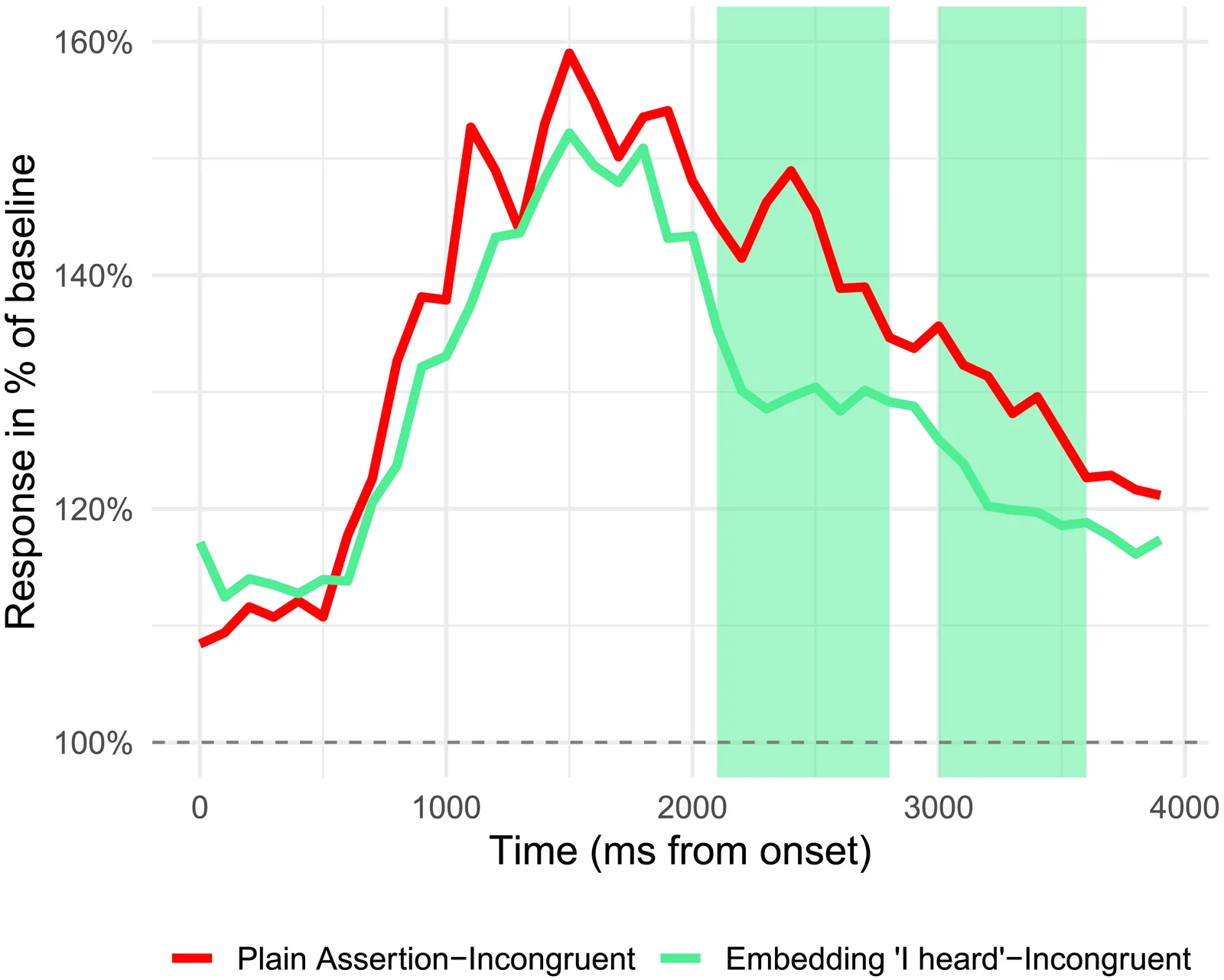
Significant clusters of plain assertions versus embedding ‘I heard’ items.

**Figure 7.**
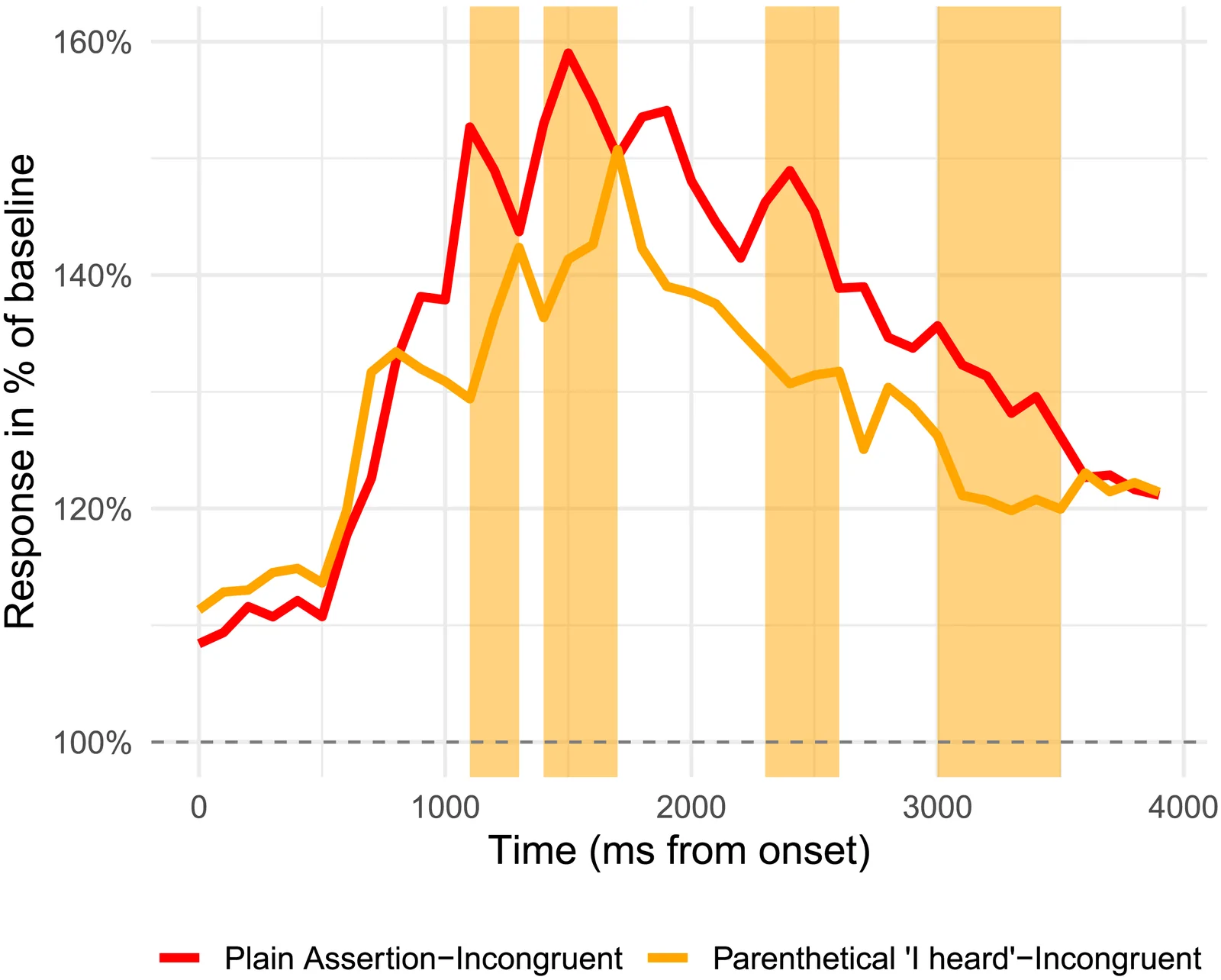
Significant clusters of plain assertions versus parenthetical ‘I heard’ items.

Finally, comparing the embedding ‘i heard’–incongruent and parenthetical ‘i heard’–incongruent conditions, no significant clusters were observed. In line with the mean-based analyses, the two evidential constructions thus did not differ reliably in their temporal dynamics.

### Peak Activation

A reviewer asks whether the observed faster return towards baseline fully accounts for the observed difference in mean activation between plain incongruent assertions and the two ‘I heard’ conditions, or whether the conditions also differ earlier, e.g., in the height of peak activation.

To investigate this, we carried out an unplanned analysis of peak activations in the critical segment. We fit a linear mixed-effects regression model predicting the per-trial 95% percentile of corrugator activation during the critical segment (expressed as a percentage of baseline). The model included fixed effects of condition and trial number, as well as random intercepts for participants and items. We used the same Helmert-style contrasts as in the analyses of Hypotheses (i) and (ii). We analysed the 95% percentile rather than the absolute maximum because the latter is highly sensitive to noise and outlying observations.

The analysis indicated that peak activation was marginally higher for the plain incongruent condition than for the two ‘I heard’ conditions (*b* = 17.33, *SE* = 9.04, *p* = .06). The difference between the two ‘I heard’ conditions was not significant (*b* = −1.78, *SE* = 10.44, *p* = .86).

Taken together, these two exploratory analyses suggest that the difference between plain incongruent assertions and the two evidential conditions reflects both a more sustained corrugator response and, potentially, somewhat higher peak activation in the plain assertion condition. However, the evidence for differences in peak activation is only marginal and should therefore be interpreted with caution.

### Filler Items

The filler items were included to determine whether differences in corrugator activation could be attributed to the syntactic structure of the constructions rather than to their evidential meaning. If the embedding and parenthetical structures differ from plain assertions in processing difficulty or complexity, such differences might be expected to emerge even in filler items that do not involve conflicting discourse commitments.

Figures 8 and 9 show the results for the filler conditions. We fit a linear mixed-effects model predicting mean activation over the critical segment, with fixed effects of syntactic condition (embedding vs. parenthetical) and the presence of a contrastive marker (with vs. without) and random intercepts for participants and items. Neither syntactic condition (*b* = −0.19, *SE* = 2.13, *t* = −0.09, *p* = .93, 95% CI = [−4.37, 3.99]) nor the presence of a contrastive marker (*b* = 0.25, *SE* = 2.13, *t* = 0.12, *p* = .91, 95% CI = [−3.93, 4.42]) had a significant effect on corrugator activation. Corrugator activity remained close to baseline throughout the filler items. These results suggest that the differences observed in the experimental conditions are unlikely to be driven by the syntactic properties of the constructions themselves, and instead are more plausibly related to the discourse-level manipulation of speaker commitment.

**Figure 8.**
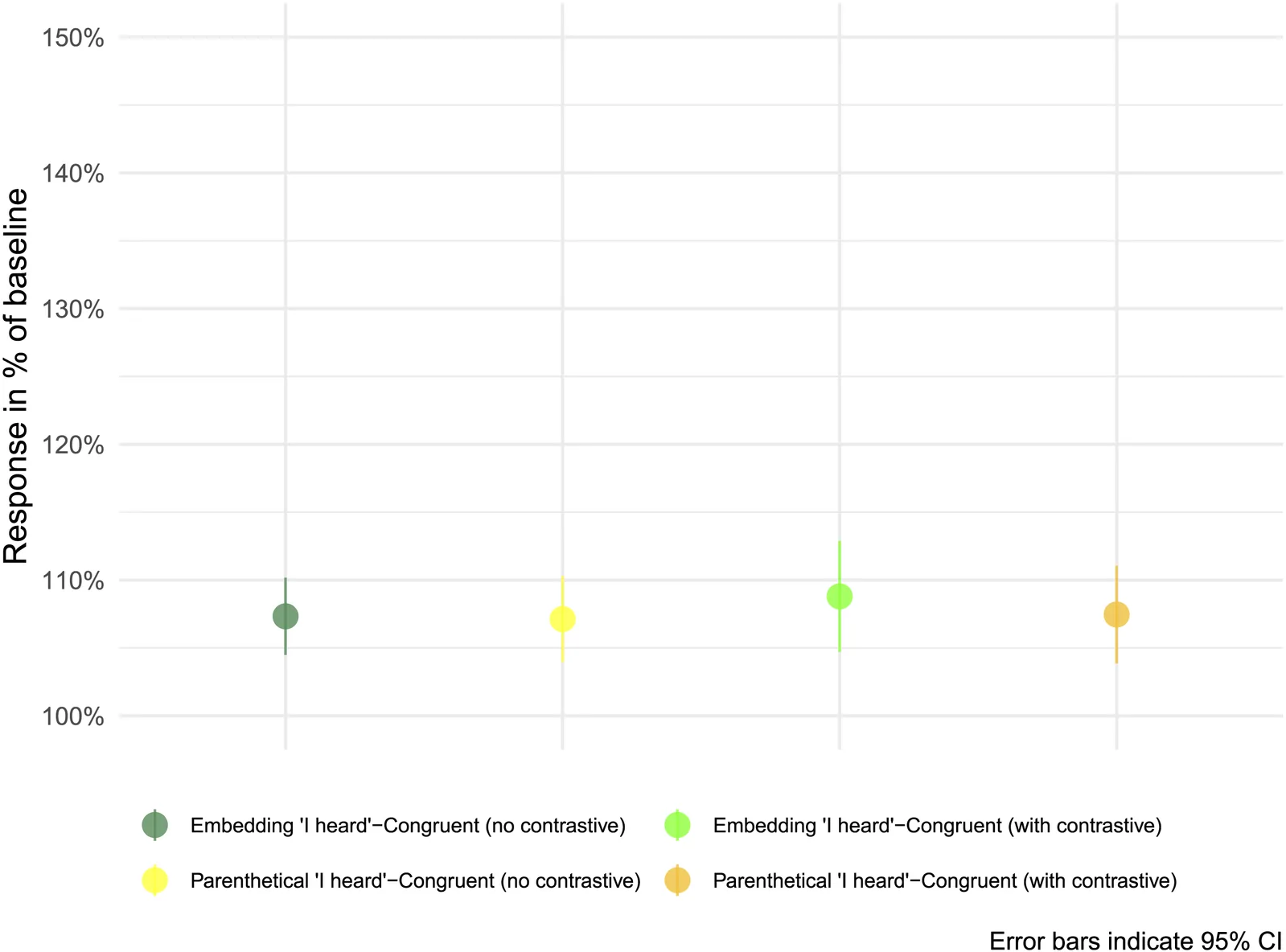
By-part means plot of corrugator activation throughout critical segment in filler conditions.

**Figure 9.**
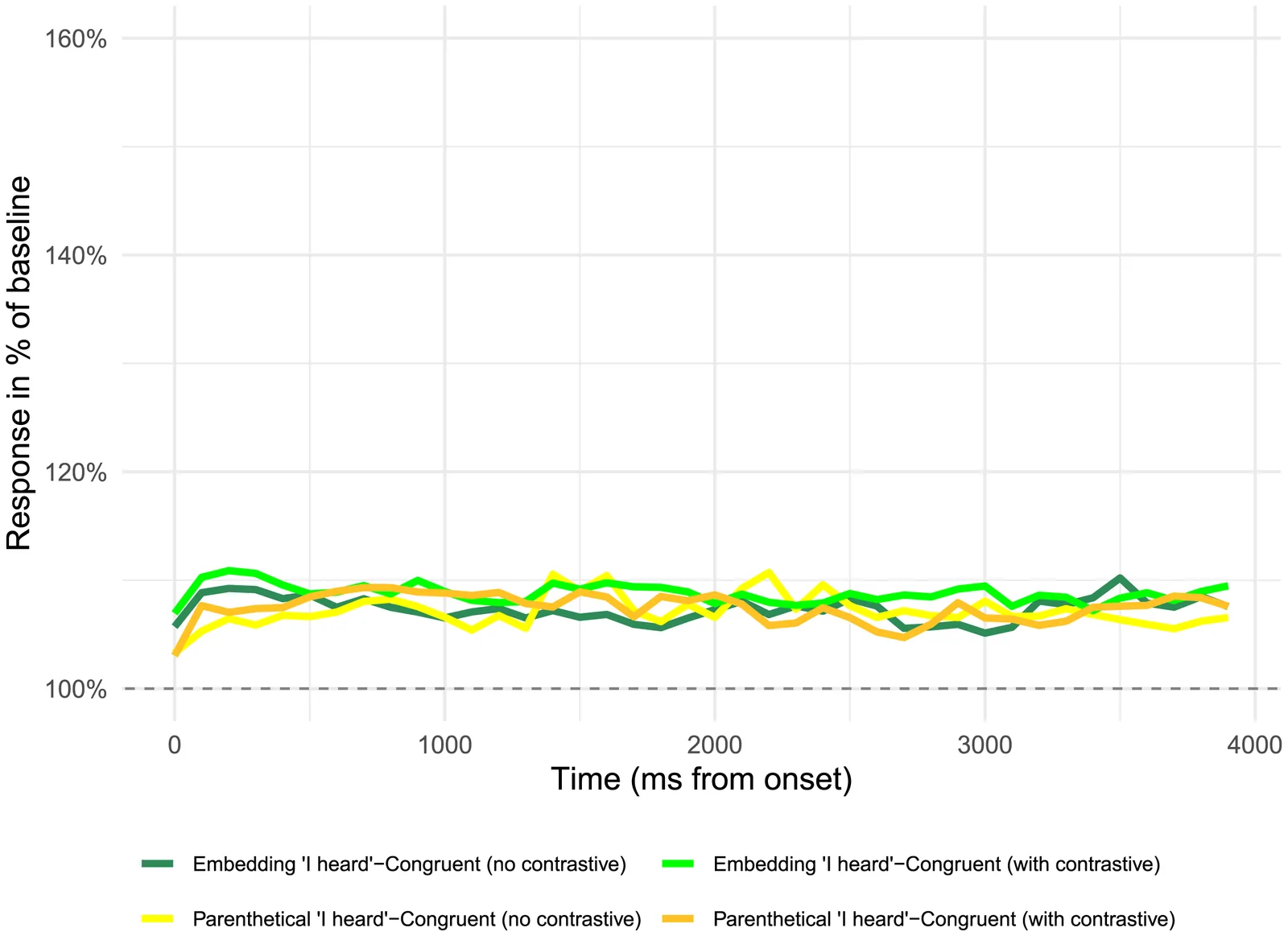
Timeplot of corrugator activation over critical segment in filler conditions.

## DISCUSSION

### Interpretation of Findings

In order to understand the implications of the results, it is important to revisit the rationale for using fEMG in this study. As Bartholow et al. (2001) have shown, the observation of norm violations elicits corrugator activity. In the context of this study, the relevant norm is that a speaker should not act in conflict with their prior discourse commitments. Thus, the working hypothesis of this study is that greater corrugator activation reflects a stronger perceived violation of speaker commitment.

As illustrated in Figure 3, corrugator activity remained relatively low and stable throughout the trial period until the critical follow-up segment. This suggests that participants did not perceive unintended pragmatic norm violations during either the story introduction or the initial move segment. Moreover, the absence of elevated corrugator activity during the initial move segment makes it unlikely that the presence of ‘I heard’ or its syntactic realization, by itself, triggered frowning responses.

Similarly, the plain assertion–congruent control condition (as well as the filler conditions) elicited minimal corrugator activation during the critical follow-up segment, as expected given that the speaker does not violate any prior discourse commitments. In contrast, the plain assertion–incongruent condition, which involves an explicit commitment violation, elicited significantly elevated corrugator activity relative to the control condition. These findings align with the results of the prior proof-of-method study (Yates et al., 2025), and provide further support for the reliability of our measurement approach.

The ‘I heard’ conditions also elicited significantly greater corrugator activation than the control condition, though not to the same extent as the plain assertion–incongruent condition. When a speaker used ‘I heard’ to indicate hearsay evidence for a proposition and subsequently contradicted that proposition, corrugator activity was significantly lower than when a plain assertion was contradicted. On the working hypothesis that greater corrugator activation reflects a stronger perceived commitment violation, these results suggest that plain assertions carry greater commitment to the expressed proposition than reportative statements marked with ‘I heard’, contra option (b) in the introduction. At the same time, the reportative conditions still elicited elevated corrugator activity relative to the congruent control condition, suggesting that contradictions of reportative statements are not perceived as entirely commitment-free. That makes option (a) less plausible as well. According to that option, the content introduced with Dutch ‘ik hoorde’ is just *presented* (that is, just put on the table for discussion by the speaker without any commitment whatsoever), as, for example, Faller (2002) proposes for the Cuzco Quechua reportative evidential –*si*.^3^ Instead, our results support option (c): the reportative evidential reduces, but does not altogether remove, the speaker’s commitment.

To gain a better understanding of the observed difference in mean corrugator activation, we further analyzed the temporal dynamics of the response. These exploratory analyses indicate how the difference between plain assertions and reportative statements is reflected in the temporal profile of corrugator activity. Specifically, the cluster-based analyses indicated that the largest condition differences occurred towards the end of the critical segment, with both reportative conditions showing a faster return towards baseline than the plain assertion condition. In addition, an exploratory and unplanned analysis of peak activation provided tentative evidence that the plain assertion condition elicited higher peak activation than the ‘I heard’ conditions, although this effect was only marginally significant.

Because these analyses were exploratory and were not designed to test a specific hypothesis, they should be interpreted with caution. While they suggest that the conditions differ not only in overall activation but also in the temporal dynamics of the response, the present data do not allow us to determine precisely which aspects of the response give rise to the observed mean differences. Future studies specifically designed to investigate these temporal dynamics are needed to establish how commitment-related processing unfolds over time.

Although the exploratory analyses do not by themselves establish the mechanism underlying these temporal differences, they nevertheless suggest an interesting possibility. In particular, the finding that the reportative conditions returned more rapidly towards baseline is consistent with the idea that hearers initially interpret reportative statements as involving a relatively strong commitment to the reported content, but subsequently revise this interpretation when confronted with a contradiction.

One way of developing this idea is provided by the account of speech reports and commitment proposed by Bary and Maier (2021). On this view, a reportative sentence introduces the reported content under the scope of an epistemic necessity operator whose modal base is determined by context. A hearer may initially adopt a relatively strong interpretation, roughly corresponding to “for all the speaker knows”. If such a statement is immediately followed by a denial of the reported content, this may initially generate a substantial expectation violation and hence a strong corrugator response. However, unlike a plain assertion, a reportative statement also permits weaker interpretations in which the relevant modal base is restricted to information obtained through hearsay. Once the hearer recognizes that such an interpretation is available, the apparent conflict may be partially resolved.

On this speculative interpretation, the elevated corrugator activity observed for the reportative conditions reflects an initial reaction to an apparent commitment violation, whereas the more rapid decline in activation reflects the hearer’s adjustment to a weaker interpretation of the speaker’s commitment. By contrast, no comparable reinterpretation is available in the case of a plain assertion, which may explain why corrugator activation remains elevated for longer. Importantly, the present data do not directly test this account. Rather, the account is offered as one possible explanation of the temporal patterns observed in the exploratory analyses.

These findings suggest a discourse framework that models not only the contents of what is said but also the degree of commitment to these contents. Inspiration for such a model can be found in Northrup’s (2014) proposal where an update of a speaker’s discourse commitments consists of a pair of the propositional content and the evidential base.

No significant differences in corrugator activity were observed between embedding (‘I heard that *p*’) and parenthetical (‘*p*, I heard’) constructions. Thus, we found no evidence that the syntactic prominence of the propositional content plays a significant role in the attribution of a speaker’s commitment.

### Limitations and Future Directions

Having outlined the main findings, we must also consider some possible limitations of this study.

A general limitation of the study is its linguistic scope. The experiment studied Dutch ‘ik hoorde’, which means that, in a strict sense, the conclusions apply to this construction in this language. Other reportative expressions in Dutch or other languages may well behave differently. Even a seemingly small change, such as from past tense ‘ik hoorde’ to present tense ‘ik hoor’, may well lead to different results. This limitation also entails that our study cannot be interpreted as falsifying claims in the literature about other reportative constructions or other languages. Although our findings at first sight seem to go against Hunter (2016), who claims that in the parenthetical construction the degree of commitment is the same as in the plain assertion, or against Murray (2014) who claims that in the embedding construction there is no commitment, their claims are about English ‘she said’ and ‘I heard’, respectively.

In addition, our study investigates how people *perceive* certain speech acts. This is the result of a combination of factors, of which the semantics of the reportative construction, which Murray focuses on, is only one element. More research is needed to investigate factors such as tense, Question-Under-Discussion, the perceived reliability of the information source (Koev, 2021), and the grammatical realization of the reportative expression (verb, clitic, modal). This study developed a method that can be applied in these future studies.

A second limitation is its demographic scope. It should be noted that, due to the University-centralized recruitment method, the participant sample consisted predominantly of individuals with university-level education, primarily of Dutch nationality, resulting in a relatively homogeneous WEIRD (i.e., Western, Educated, Industrialized, Rich, and Democratic) population (Henrich et al., 2010). This may raise questions about the generalization of the observed effects to populations with different sociocultural backgrounds, communicative practices, or patterns of emotional expression. Future research should investigate whether similar patterns persist in different populations.

We must also be aware of what this study should not be taken as. The results fit a gradable picture on commitment, and are certainly suggestive. Still, it would not be justified to take this experiment as proving a gradable notion of commitment in the strictest sense of the word. The alternative, a black-or-white view on commitment, is often presupposed in more formal and more philosophical work (e.g., Geurts, 2019; Singh, 2000). Although the results fit a gradable view very well, it is not excluded that they are in principle also compatible with a view where commitments themselves are categorial but what evidential markers modulate are the *objects* of commitments. A ‘reduced commitment’ could then mean a (full) commitment to ‘possibly *p*’. More research is needed on this.

While fEMG is highly sensitive to subtle affective responses, it may have limits in detecting the most nuanced degrees of commitment. Our study’s design—with a large sample and many items—was sufficiently sensitive to detect the key theoretical distinction: reduced corrugator activity for evidentials versus plain assertions. We do not interpret null results (e.g., between evidential constructions) as evidence of no difference, but they might suggest such fine-grained distinctions may not manifest in this measure. The positive findings, however, reliably capture a core aspect of participants’ understanding.

Looking ahead to future work, it would be beneficial to explore the nuances of temporal data in more detail. In particular, implementing word-based time-locking could yield more precise insights into how responses unfold relative to specific linguistic events. Such precise temporal mapping is a valuable aspect of fEMG which we have not fully utilised in the present study. Whereas the current study offers a useful insight into overall response trajectories across the 4,000 ms window of the follow-up utterance, and an exploratory temporal analysis, future work distinguishing individual word onsets would allow researchers to pinpoint when within an utterance critical differences emerge. This would help clarify potential word-level dynamics that may not be fully captured by broader segment-level analyses.

Given the contributions made in this study, we maintain that fEMG is a valuable tool in exploring commitment negotiation in communication. Importantly, its sensitivity to subtle, graded affective responses suggests that fEMG can capture not only clear-cut contrasts as explored in previous work, but also the nuanced distinctions that are crucial for understanding real-time communication. Moving beyond reportative evidentiality, fEMG can be applied to more questions regarding commitments in communication. We could for example use the methodology to compare the perceived commitment attributed to speakers who assert, implicate or presuppose certain content (Mazzarella et al., 2018). Further options are to investigate the contribution of gestures or intonation, or the commitments of other conversational participants, such as the addressee. Moving beyond commitments to the truth of a proposition, we next aim to investigate ‘commitments de lingua,’ that is, a speaker’s commitment to the appropriateness of their specific word choice. In such experiments, it will be a great asset to time-lock stimuli on a word-by-word basis rather than across entire phrases, thus advancing our understanding of how communicative commitments are managed in natural discourse.

### Conclusion

This study demonstrates that a speaker’s use of hearsay markers does influence the perception of the speaker’s commitment to their utterances. When contradicting themselves, speakers who initially presented propositional content in the form of a plain assertion trigger more frowning from observers than speakers who explicitly marked their proposition as reportative. There was no evidence that the embedding vs. parenthetical variation has a significant effect. The findings suggest a gradable notion of commitments, wherein reportative markers impact the degree of perceived speaker commitment.

These findings also underscore the suitability of fEMG as a method for capturing such nuanced pragmatic distinctions, beyond generally agreed-upon cases. We advocate for the use of fEMG as an experimental method in pragmatics and philosophy of language, as others have advocated for the methodology’s value in language comprehension (van Berkum et al., 2023), social psychology (Bartholow et al., 2001), and political science (Schumacher et al., 2024). Our study has shown that fEMG is a promising method to progress our understanding of commitment attribution, negotiation, and avoidance in conversation, which constitute the foundation of our social lives.

## ACKNOWLEDGMENTS

We would like to thank our student intern, Iga Maśniak, for her extremely valuable work on data collection throughout this experiment.

## FUNDING INFORMATION

This publication is part of the project Commitments in Communication with file number 406.22.24CTW.026 of the research programme Open Competition M which is financed by the Dutch Research Council (NWO) under the grant https://doi.org/10.61686/SYQRZ19926.

## AUTHOR CONTRIBUTIONS

H.Y.: Conceptualization; Data curation; Formal analysis; Investigation; Methodology; Project administration; Software; Validation; Visualization; Writing – original draft; Writing – review & editing. B.v.T.: Conceptualization; Data curation; Formal analysis; Methodology; Software; Supervision; Validation; Visualization; Writing – original draft; Writing – review & editing. P.d.S.: Conceptualization; Formal analysis; Methodology; Software; Supervision; Validation; Visualization; Writing – original draft. C.B.: Conceptualization; Funding acquisition; Methodology; Project administration; Supervision; Writing – original draft; Writing – review & editing.

## DATA AVAILABILITY STATEMENT

For all materials related to this study including item lists, analysis scripts, and full data output, see OSF repository: https://osf.io/uzd6h.

## Notes

^1^ For readability we will refer to ‘I heard’ rather than the Dutch original, without intending to imply that the two are exactly the same.^2^ See, for example, Jackendoff (1972) for ‘I think’, ‘we understand’, ‘I fear’, Asher (2000) for ‘she assured us’, Hunter (2016) for ‘she said.’^3^ Less plausible although not entirely impossible: for example, Faller could contest that participants react emotionally to the fact that the speaker was apparently told incorrect information (and feels vicariously indignant). Or perhaps that presenting something leads to discourse expectations, even when not endorsed.
